# Feasibility of ipsilateral lower neck sparing irradiation for unilateral or bilateral neck node-negative nasopharyngeal carcinoma: systemic review and meta-analysis of 2, 521 patients

**DOI:** 10.1186/s13014-018-1087-x

**Published:** 2018-08-06

**Authors:** Cheng-Long Huang, Cheng Xu, Yuan Zhang, Guan-Qun Zhou, Yan-Ping Mao, Qing Liu, Ying Sun, Jun Ma, Ling-Long Tang

**Affiliations:** 1 Department of Radiation Oncology, Sun Yat-sen University Cancer Center; State Key Laboratory of Oncology in South China; Collaborative Innovation Center for Cancer Medicine; Guangdong Key Laboratory of Nasopharyngeal Carcinoma Diagnosis and Therapy, Guangzhou, 510060 China; 2 Department of Medical Statistics and Epidemiology, School of Public Health, Sun Yat-sen University, Guangzhou, People’s Republic of China

**Keywords:** Nasopharyngeal carcinoma, Lymph node metastases, Ipsilateral lower neck sparing irradiation, Ipsilateral lower neck prophylactic irradiation, Survival

## Abstract

**Background:**

To compare the efficacy of ipsilateral lower neck sparing irradiation (ILNSI) versus ipsilateral lower neck prophylactic irradiation (ILNPI) for unilateral or bilateral neck node-negative nasopharyngeal cancer (NPC).

**Methods:**

A comprehensive literature search of PubMed, EMBASE, the Cochrane Library and other public databases was conducted in October, 2017. The outcomes were 3-year overall/regional recurrence-free/disease-free/distant metastasis-free survival (OS/RRFS/DFS/DMFS) and ipsilateral lower neck (ILN) recurrence. We performed subgroup analysis of ILNSI versus ILNPI for different radiotherapy techniques. Sensitivity analysis was performed to examine the stability of the results.

**Results:**

Nine head-to-head comparative studies (2, 521 patients) were included in the meta-analysis. For the comparison of ILNSI versus ILNPI, there was no significant difference in 3-year OS (HR = 1.16, 95% confidence interval [CI] = 0.85–1.58, *P* = 0.36), RRFS (HR = 1.37, 95% CI = 0.76–2.47, *P* = 0.30), DFS (HR = 1.08, 95% CI = 0.80–1.44, *P* = 0.62) and DMFS (HR = 1.00, 95% CI = 0.69–1.44, *P* = 0.99). ILNSI and ILNPI also led to equivalent ILN recurrence rates (OR = 0.98, 95% CI = 0.47–2.03, *P* = 0.96). No significant heterogeneity was observed for any outcome. Subgroup analysis confirmed no significant differences between ILNSI and ILNPI for any outcome, regardless of radiotherapy technique. Sensitivity analysis indicated all outcomes were highly stable in favor of the original conclusions.

**Conclusions:**

ILNSI provided equivalent survival outcomes and regional control compared to ILNPI; ILNSI represents an appropriate alternative strategy for patients with unilateral or bilateral neck node-negative NPC.

**Electronic supplementary material:**

The online version of this article (10.1186/s13014-018-1087-x) contains supplementary material, which is available to authorized users.

## Background

Nasopharyngeal carcinoma (NPC) is endemic in Southeast Asia, especially in Southern China where the yearly incidence is 30 to 80 per 100,000 [1]. Since the nasopharyngeal lymphatic network is well-developed, cervical lymph node (LN) metastases are very common in NPC. Approximately 75% of patients present with enlarged neck node(s), an important physical sign, at diagnosis [2]. With the widespread use of advanced imaging methods such as magnetic resonance imaging (MRI) and positron emission tomography/computed tomography (PET/CT), LN metastases can now be detected more easily and accurately. Around 85% of patients have LN metastasis during initial staging [3]. Radiotherapy has definitive therapeutic efficacy for NPC and is therefore the primary treatment [4]. About 30% patients with neck node-negative NPC who do not receive neck irradiation subsequently develop LN involvement [5]. Thus, the radiation target routinely includes the primary tumor, the retropharyngeal area and the whole neck (levels II–V), regardless of nodal status [6]. However, in this mode of irradiation, the incidence of irradiation-induced hypothyroidism is up to 22 to 29% [7, 8].

Several recent studies have reported LN metastasis spreads in an orderly fashion from the higher to lower level LNs in NPC, and skip metastases are rare [9]. The retropharyngeal lymph nodes (RLNs) and level II LNs are the most frequently involved LNs [10]. Thus, elective neck irradiation could represent an alternative clinical strategy that may not impair regional control or survival outcomes [11]. Several studies have reported elective 50–56 Gy irradiation of the upper neck including the level II, III and VA LNs is suitable and advised for patients with neck node-negative NPC [11]. Moreover, compared with whole neck irradiation, omitting irradiation of the lower neck reduces irradiation-induced adverse reactions, such as hypothyroidism [12]. However, Fu et al. indicated lower neck irradiation is necessary for N0 NPC if the tumor volume, which is positively associated with LN metastases, is greater than 10 mL [13]. Therefore, the necessity of lower neck irradiation for unilateral or bilateral neck node-negative NPC is still unclear, and worthy of further investigation.

This meta-analysis aimed to thoroughly investigate and compare the efficacy of ipsilateral (on the same side as a unilateral node-negative neck, or both sides of a bilateral node-negative neck) lower neck (the extension below the caudal border of the cricoid cartilage) sparing irradiation (ILNSI) and ipsilateral lower neck prophylactic irradiation (ILNPI) in terms of survival outcomes and regional control in unilateral or bilateral neck node-negative NPC.

## Methods

A prospective protocol covering objectives, study selection, outcomes of interest and statistical analysis methods was initially planned according to the Preferred Reporting Items for Systematic Reviews and Meta-analysis (PRISMA) and Meta-Analysis of Observational Studies in Epidemiology recommendations for study reporting (MOOSE) [14, 15].

### Identification and eligibility of relevant studies

A literature search was performed of electronic databases, including PubMed, EMBASE, Cochrane Library and other public databases, in October, 2017. The following keywords in all possible multiple combinations were searched in [Title/Abstract]: nasopharyngeal carcinoma/nasopharyngeal cancer/nasopharyngeal neoplasm/NPC, irradiation/radiotherapy/radiation/emission. To broaden the search, the ‘Related Articles’ function was used; manual searching of the reference lists of included studies was also performed.

The studies included met all of the following pre-defined criteria: (1) studies that compared ILNSI with ILNPI in unilateral or bilateral neck node-negative NPC; (2) patients with newly diagnosed, pathologically confirmed and untreated NPC; (3) studies reporting at least one of the following outcomes: survival data (overall survival [OS], regional recurrence-free survival [RRFS], disease-free survival [DFS] and distant metastases-free survival [DMFS]) and LN recurrence; (4) when multiple studies focused on the same population, the study reporting the most detailed data was used; (5) the number of patients enrolled in this study was not restrained. Editorials, letters to editors, reviews, case reports, basic research reports and conference abstracts were excluded.

### Data extraction

Using a standardized data extraction form, two investigators (C.L.H. and C.X.) independently extracted data from all included studies, including first author, year of publication, study design, sex, age, detailed tumor-node-metastasis staging, pathology, main imaging methods, region of neck irradiation, number of patients receiving ILNSI or ILNPI, number of nodal recurrence events, time-to-event data (survival outcomes) and side effects. Hazard radios (HRs) were extracted directly or indirectly calculated [16]. Two investigators (C.L.H. and C.X.) examined the accuracy of the data extracted from each individual study, and any discrepancies were settled in collaboration with a senior professor (L.L.T.).

### Quality assessment

The methodological quality of randomized controlled trials (RCTs) was assessed using the Cochrane risk of bias tool, which includes six factors: random sequence generation, allocation concealment, performance bias, blinding of assessment, attrition, and reporting bias [17]. Methodological quality of retrospective studies was assessed with the modified Newcastle-Ottawa scale, which consists of three factors: patient selection, comparability of the study groups, and assessment of outcomes [18, 19]; a scale ranging from 0 to 9 was used to evaluate study quality except for RCTs. RCTs and retrospective studies with scores ≥8 were regarded as high-quality. Two investigators (C.L.H. and C.X.) independently rated all studies; the *kappa* coefficient was calculated to evaluate the agreement of the two investigators’ assessment of study quality, with *P*-values < 0.05 indicating good agreement.

### Statistical analysis

The outcomes were OS, RRFS, DFS, DMFS and ILN recurrence. Beginning from day 1 of treatment, OS was defined as the time to the date of death due to any cause or latest known date alive; RRFS, to regional recurrence; DFS, to failure, death from any cause or last follow-up visit, whichever occurred first; DMFS, to distant failure. ILN recurrence was diagnosed by MRI, CT or palpation during follow-up. The meta-analysis, forest plots and funnel plots were developed using Review Manager 5.2 (Cochrane Collaboration, Oxford, UK).

Hazard ratios (HRs) and 95% confidence intervals (CIs) were used as summary statistics for time-to-event data (OS, RRFS, DFS and DMFS) [20]. Odds ratios (ORs) and 95% CIs were used to measure dichotomous variates (ILN recurrence rate). An OR or HR < 1 represented a benefit favoring ILNSI; if the upper limit of a 95% CI was less than 1, the benefit of ILNSI was statistically significant (*P* < 0.05). According to the guidelines by Parmar, the pooled HRs and 95% CIs were calculated from the natural logarithm of HR (lnHR) and standard error of the lnHR (se[lnHR]) [21]. Statistical heterogeneity between studies was assessed using the *χ*^*2*^ and *I*^*2*^ tests, with significance set at *P* < 0.10. Heterogeneity was regarded as high, moderate or low if *I*^*2*^ > 75, 50% or 25%, respectively [22]. If significant heterogeneity existed, the random-effects model was used; if not, we used the fixed-effects model [23].

Subgroup analysis of ILNSI versus ILNPI was performed to further investigate the feasibility of ILNSI according to different radiotherapy techniques, i.e., two-dimensional radiotherapy (2D-RT) and IMRT. To test the stability of the results, sensitivity analysis of the comparison for ILNSI versus ILNPI that included English studies, high-quality studies, studies matched for T category, and studies detecting LN metastases mainly via MRI were performed. To detect potential publication bias in the funnel plots, Egger’s tests were performed using Stata software 12.0 (StataCorp, College Station, TX, USA). A *P*-value < 0.05 indicated significant publication bias.

## Results

### Characteristics of eligible studies

**Fig. 1:**
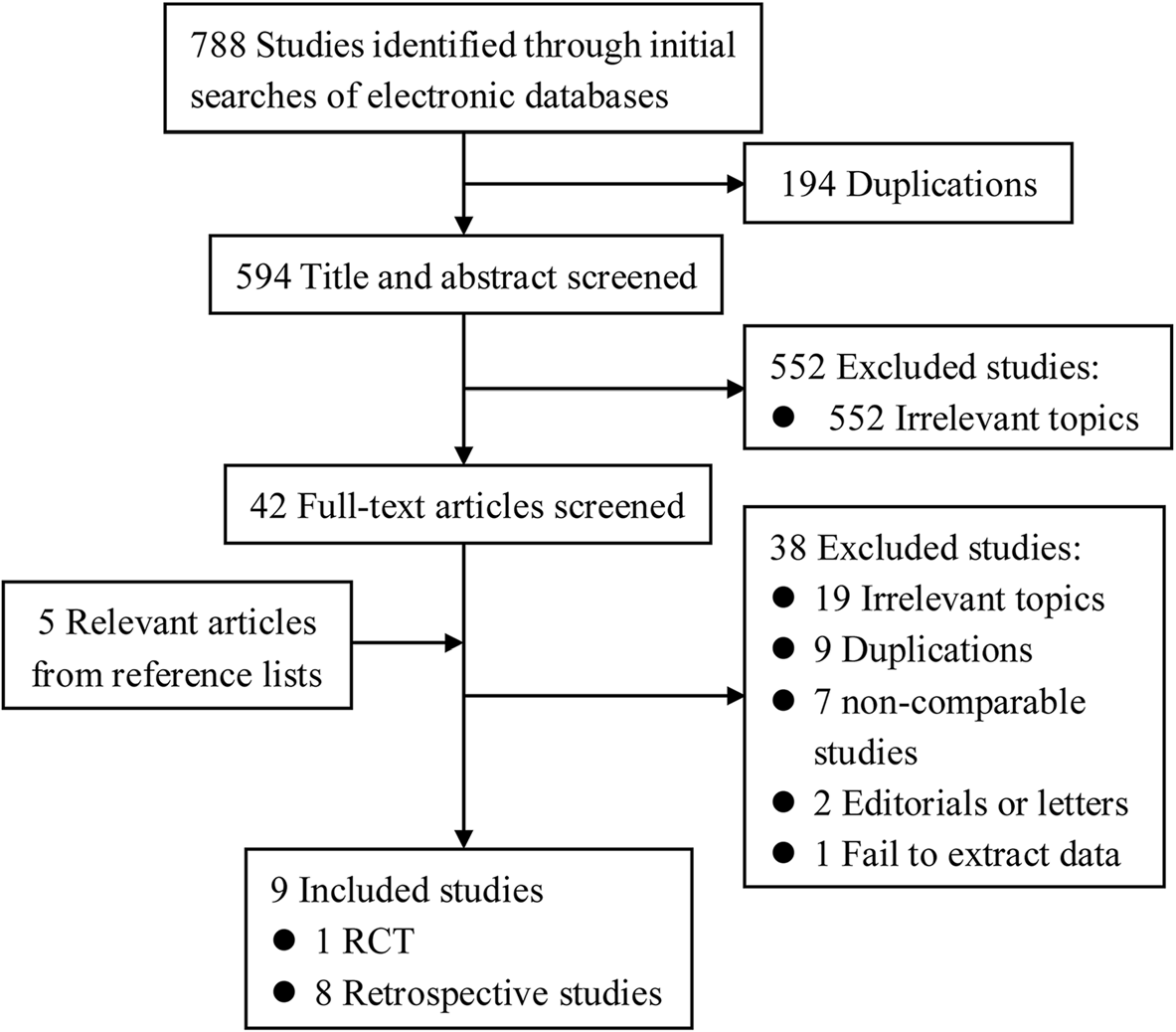
Flow diagram of study identification, inclusion and exclusion. RCT: randomized controlled trials

As shown in Fig. 1, nine studies, including 2, 521 patients (937 patients for ILNSI and 1, 584 patients for ILNPI), fulfilled the pre-defined criteria and were included in the final analysis [10, 24–31].

The baseline characteristics of the nine included studies are shown in Table 1. Eight of the nine included articles were retrospective studies [10, 24–26, 28–31]; the other was a RCT [27]. Seven studies were published in English [10, 24–29], and two studies were in Chinese with English abstracts [30, 31]. All studies included patients with unilateral or bilateral neck node-negative NPC receiving ILNSI or ILNPI during 1989–2012. Seven studies assessed LN metastasis by imaging methods such as MRI and/or CT [10, 24–27, 29, 30]; the remaining two studies, by palpation [28, 31]. The summary of radiotherapy parameters of the included studies is shown in Table 2. Three studies used IMRT [24–26], two studies used 2D-RT [30, 31], and the remaining studies used 2D-RT, IMRT and three-dimensional conformal radiotherapy (3D-CRT) [10, 27–29]. Quality assessment of the included eight retrospective studies and one RCT is fully reported in Additional file 1: Tables S1 and S2, respectively. The single RCT and five retrospective studies were identified as high-quality with scores ≥8 [24, 26–30], while the three retrospective studies were identified as relatively low-quality [10, 25, 31] (Additional file 1: Figures S1 and S2). The *kappa* coefficient was 80.00% (*P* = 0.001).

**Table 1:** Baseline characteristics of the nine included studies

First author /year	Time range	Design	No. of patients (ILNSI/ ILNPI)	Matching items^a^	Method of assessing LN metastasis	N category	Histologic type (WHO)	Clinical stage	Median FU (range), mo.	Score
Li/2013 [27]	2005–2012	RCT	153/148	1, 2, 4, 9	MRI/CT	N0	II-III	I-IVa (AJCC 6th)	39.0 (6.0–84.0)	RCT
Chen/2014 [26]	2003–2007	R	54/100	1, 2, 4, 6, 8, 10	MRI	N1	I-II	II-IVa (AJCC 7th)	60.7 (12.2–98.9)	8
Xie/2010 [30]	2002–2004	R	88/117	1, 2, 4, 10	CT/MRI/PET/ CT	N0	NR	I-Iva (UJCC 6th)	44.0 (3.0–68.0)	8
Ou/2012 [29]	2005–2009	R	89/30	1, 2, 3, 7, 9, 10	MRI	N0	I-II	I-IV (AJCC 6th)	36.6 (8.1–76.9)	9
Tang/2017 [24]	2009–2012	R	189/357	1, 2, 4, 5, 7, 8, 10	MRI	Unilateral neck node metastasis	I-III	II-IV (UICC/AJCC 6th)	44.9 (1.3–69.2)	8
Zeng/2014 [25]	2003–2008	R	171/99	1, 2, 3, 4, 6, 10	MRI/PET/CT	N0	II-III	NR	65.1 (4.0–106.0)	7
Li/2005 [31]	1997–1998	R	88/90	1, 2, 4, 10	Palpation	N0	I	I-Iva (1992 FCSS)	NR	6
Tang/2009 [10]	2003–2004	R	37/101	NR	MRI	N0	I-III	I-IV (AJCC 6th)	43.0 (2.0–59.0)	6
Sun/2012 [28]	1989–2009	R	68/542	1, 2, 4, 8, 9, 10	Palpation	N0	I-III	NR	85.0 (3.0–254.0)	8

*Abbreviations*: *no.* number; *ILNSI* ipsilateral lower neck sparing irradiation, *ILNPI* ipsilateral lower neck prophylactic irradiation, *LN* lymph nodes, *FU* follow-up, *MRI* magnetic resonance imaging, *CT* computed tomography, *PET/CT* positron emission tomography/computed tomography, *WHO* World Health Organization, *AJCC* American Joint Committee on Cancer, *UICC* Union for International Cancer Control, *FCSS* FuZhou Chinese Staging System, *mo.* month, *RCT* randomized controlled trial, *R* retrospective, *RLN* retropharyngeal lymph nodes, *NR* not reported

^a^ Matching items include:1 = sex, 2 = age, 3 = pathology, 4 = T category, 5 = N category, 6 = features of RLN, 7 = stage, 8 = histologic type (WHO), 9 = radiotherapy technique, 10 = chemoradiotherapy

**Table 2:** Summary of radiotherapy parameters for the included studies

Study/year	Arms	Technique	Radiotherapy parameters			
			GTVnx	GTVnd	CTV1	CTV2
Li/2013 [27]	Arms1&2	2D-RT, IMRT	70Gy (2Gy/fx)	50Gy (2Gy/fx)	CTV: 70Gy (2Gy/fx)	
Chen/2014 [26]	Arms1&2	IMRT	68Gy (2.27Gy/fx/d, 5fx/wk)	54Gy (1.8Gy/fx/d, 5fx/wk)	60Gy (2Gy/fx/d, 5fx/wk)	54Gy (1.8Gy/fx/d, 5fx/wk)
Xie/2010 [30]	Arm1	2D-RT	71.64Gy	51.8Gy	NR	NR
	Arm2	2D-RT	72.52Gy	52.26Gy	NR	NR
Ou/2012 [29]	Arm1	2D-RT, 3D-CRT, IMRT	GTV: 66.0–70.4Gy/30-32fx;		CTV: 60Gy/30-32fx	
	Arm2	2D-RT	70-76Gy (2Gy/fx);	50-62Gy (1.8–2.0Gy/fx)	NR	NR
Tang/2017 [24]	Arms1&2	IMRT	66-72Gy (2.12–2.43Gy/fx)	64-70Gy/28-33fx	60-63Gy/(28-33fx)	54-56Gy/(28-33fx)
Zeng/2014 [25]	Arms1&2	IMRT	68Gy (2.27Gy/fx)	60Gy (2Gy/fx)	60Gy (2Gy/fx)	54Gy (1.8Gy/fx)
Li/2005 [31]	Arm1	2D-RT	70.1Gy (2Gy/fx/d, 5fx/wk)	52.9Gy (2Gy/fx/d, 5fx/wk)	NR	NR
	Arm2	2D-RT	70.4Gy (2Gy/fx/d, 5fx/wk)	53.4Gy (2Gy/fx/d, 5fx/wk)	NR	NR
Tang/2009 [10]	Arms1&2	2D-RT, 3D-CRT, IMRT	NR	NR	NR	NR
Sun/2012 [28]	Arms1&2	2D-RT, 3D-CRT, IMRT	70Gy (2Gy/fx/d, 5fx/wk)	50Gy (2Gy/fx/d, 5fx/wk)	CTV: 50Gy (2Gy/fx/d, 5fx/wk)	

*Abbreviations*: *2D-RT* two-dimensional radiotherapy, *IMRT* intensity-modulated radiotherapy, *3D-CRT* three-dimensional conformal radiotherapy, *GTVnx* primary gross tumor volume, *GTVnd* gross tumor volume of involved lymph nodes, *CTV1* high-risk clinical target volume, *CTV2* low-risk clinical target volume, *fx* fraction, *d* day, *wk.* week, *NR* not reported

### Survival outcomes

**Fig. 2:**
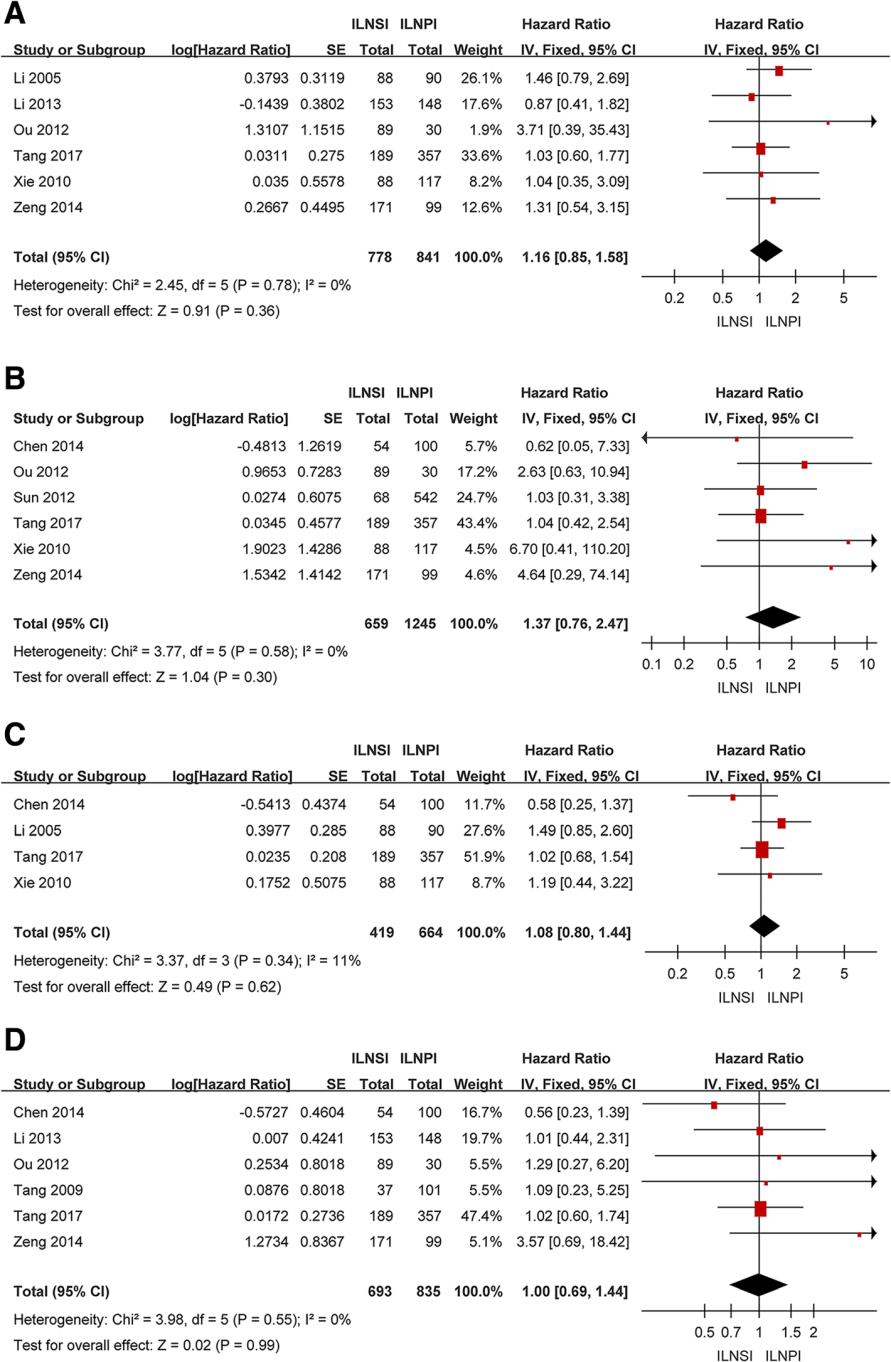
Forest plot and meta-analysis of 3-year OS (**a**), RRFS (**b**), DFS (**c**) and DMFS (**d**) following ILNSI versus ILNPI. Squares are the point estimates of the HRs with the 95% CIs indicated by horizontal bars. Diamonds are the summary estimates and 95% CIs from the pooled studies. ILNSI: ipsilateral lower neck sparing irradiation. ILNPI: ipsilateral lower neck prophylactic irradiation

As shown in Fig. 2, there was no significant difference in 3-year OS between ILNSI and ILNPI among 1, 619 patients from six studies [24, 25, 27, 29–31] (HR = 1.16, 95% CI = 0.85–1.58, *P* = 0.36); between-study heterogeneity was non-significant (*P* = 0.78). Pooled data from six studies (1, 904 patients) [24–26, 28–30] revealed no significant difference in 3-year RRFS between ILNSI and ILNPI (HR = 1.37, 95% CI = 0.76–2.47, *P* = 0.30), with non-significant between-study heterogeneity (*P* = 0.58). Pooled data from four studies (1, 083 patients) [24, 26, 30, 31] showed no significant difference in 3-year DFS between ILNSI and ILNPI (HR = 1.08, 95% CI = 0.80–1.44, *P* = 0.62); between-study heterogeneity was non-significant (*P* = 0.34). Pooled data from six studies (1, 528 patients) [10, 24–27, 29] showed no significant difference in 3-year DMFS between ILNSI and ILNPI (HR = 1.00, 95% CI = 0.69–1.44, *P* = 0.99), with no significant between-study heterogeneity (*P* = 0.55).

### ILN recurrence

**Fig. 3:**
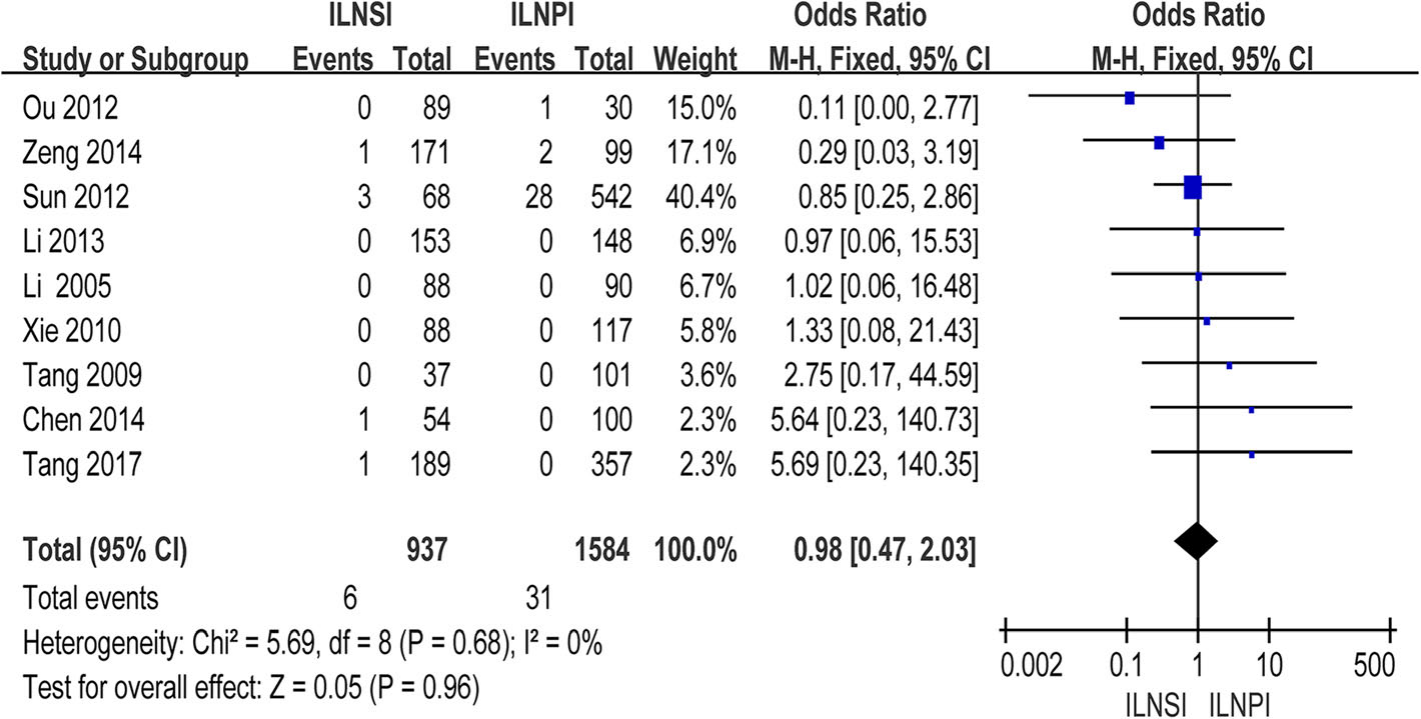
Forest plot and meta-analysis of ILN recurrence following ILNSI versus ILNPI. Squares are the point estimates of the HRs with the 95% CIs indicated by horizontal bars. Diamonds are the summary estimates and 95% CIs from the pooled studies. ILNSI: ipsilateral lower neck sparing irradiation. ILNPI: ipsilateral lower neck prophylactic irradiation

As shown in Fig. 3, pooled data from the nine studies that assessed ILN recurrence in 2, 521 patients showed no significant difference in ILN recurrence between ILNSI and ILNPI (OR = 0.98; 95% CI = 0.47–2.03; *P* = 0.96); between-study heterogeneity was non-significant (*P* = 0.68). The OR value of the single RCT was 0.97 (95% CI = 0.06–15.53) [27].

### Subgroup analysis

We excluded four studies [10, 27–29] from the subgroup analysis of radiotherapy techniques, as these studies did not report the specific techniques (i.e., IMRT or 2D-RT). Subgroup analysis including the three studies using IMRT [24–26] and two studies using 2D-RT [30, 31] yielded no significant changes in OS, RRFS, DFS, DMFS or ILN recurrence compared with the original meta-analysis (Table 3). For the subgroup analysis based on IMRT, heterogeneity between studies increased slightly for ILN recurrence (*I*^*2*^ *=* 36%, *P* = 0.21) and 3-year DMFS (*I*^*2*^ *=* 48%, *P* = 0.15), but remained non-significant (all *P* > 0.05). For the subgroup analysis based on 2D-RT, all between-study heterogeneities were non-significant, with *I*^*2*^ *=* 0% (Table 3).

**Table 3:** Subgroup analysis and sensitivity analysis of ILNSI versus ILNPI

Outcomes	No. of studies	Pts receiving ILNSI (no.)	Pts receiving ILNPI (no.)	All pts. (no.)	HR/OR (95% CI)	*P*	Study heterogeneity			
							*χ2*	df	*I*^*2*^ (%)	*P*
*Subgroup analysis*										
Studies using IMRT										
3-year OS	2	360	456	816	1.10 (0.69,1.74)	0.68	0.20	1	0	0.65
3-year RRFS	3	414	556	970	1.11 (0.50,2.49)	0.80	1.26	2	0	0.53
3-year DFS	2	243	457	700	0.92 (0.64,1.33)	0.67	26	1	26	0.24
3-year DMFS	3	414	556	970	0.97 (0.62,1.51)	0.88	3.85	2	48	0.15
ILN recurrence	3	414	556	970	1.44 (0.35,5.95) ^a^	0.62	3.12	2	36	0.21
Studies using 2D-RT										
3-year OS	2	176	207	383	1.35 (0.79,2.30)	0.27	0.29	1	0	0.59
3-year DFS	2	176	207	383	1.41 (0.87,2.30)	0.17	0.15	1	0	0.70
ILN recurrence	2	176	207	383	1.17 (0.16,8.32) ^a^	0.88	0.02	1	0	0.90
*Sensitivity analysis*										
English publications										
3-year OS	4	602	634	1236	1.07 (0.73,1.57)	0.73	1.69	3	0	0.64
3-year RRFS	5	571	1128	1699	1.27 (0.69,2.33)	0.44	2.48	4	0	0.65
3-year DFS	2	243	457	700	0.92 (0.64,1.33)	0.67	26	1	26	0.24
3-year DMFS	6	693	835	1528	1.00 (0.69,1.44)	0.99	3.98	5	0	0.55
ILN recurrence	7	761	1377	2138	0.95 (0.44,2.09) ^a^	0.91	5.64	6	0	0.46
High-quality studies										
3-year OS	4	519	652	1171	1.02 (0.69,1.52)	0.92	1.44	3	0	0.70
3-year RRFS	5	488	1146	1634	1.29 (0.71,2.37)	0.41	2.99	4	0	0.56
3-year DFS	3	331	574	905	0.95 (0.67,1.34)	0.78	1.58	2	0	0.45
3-year DMFS	4	485	635	1120	0.92 (0.62,1.36)	0.68	1.49	3	0	0.69
ILN recurrence	6	641	1291	1932	1.05 (0.46,2.42) ^a^	0.90	4.14	5	0	0.53
Studies matched for T category										
3-year OS	5	689	811	1500	1.13 (0.82,1.55)	0.44	1.41	4	0	0.84
3-year RRFS	5	570	1215	1785	1.20 (0.63,2.29)	0.54	2.81	4	0	0.59
3-year DFS	4	419	664	1083	1.08 (0.80,1.44)	0.62	3.37	3	11	0.34
3-year DMFS	5	604	805	1409	0.98 (0.67,1.44)	0.93	3.87	4	0	0.42
ILN recurrence	7	811	1453	2264	1.06 (0.49,2.33) ^a^	0.88	3.39	6	0	0.76
Studies detecting LN metastases mainly by MRI										
3-year OS	4	602	634	1236	1.07 (073,1.57)	0.73	1.69	4	0	0.64
3-year RRFS	4	503	586	1089	1.37 (0.68,2.76)	0/38	2.31	3	0	0.51
3-year DFS	4	419	664	1083	1.08 (0.80,1.44)	0.62	3.37	3	11	0.34
3-year DMFS	6	693	835	1528	1.00 (0.69,1.44)	0.99	3.98	5	0	0.55
ILN recurrence	6	693	835	1528	1.05 (0.37,2.96) ^a^	0.93	5.58	5	10	0.35

*Abbreviations*: *No.* number, *pts.* patients, *HR* hazard radio, *ILNSI* ipsilateral lower neck sparing irradiation, *ILNPI* ipsilateral lower neck prophylactic irradiation, *ILN* ipsilateral lower neck, *OS* overall survival, *RRFS* regional recurrence-free survival, *DFS* disease-free survival, *DMFS* distant metastasis-free survival, *MRI* magnetic resonance imaging, *CT* computed tomography, *IMRT* intensity-modulated radiation therapy, *2D-RT* two-dimensional radiotherapy

^a^ OR

### Sensitivity analysis

**Fig. 4:**
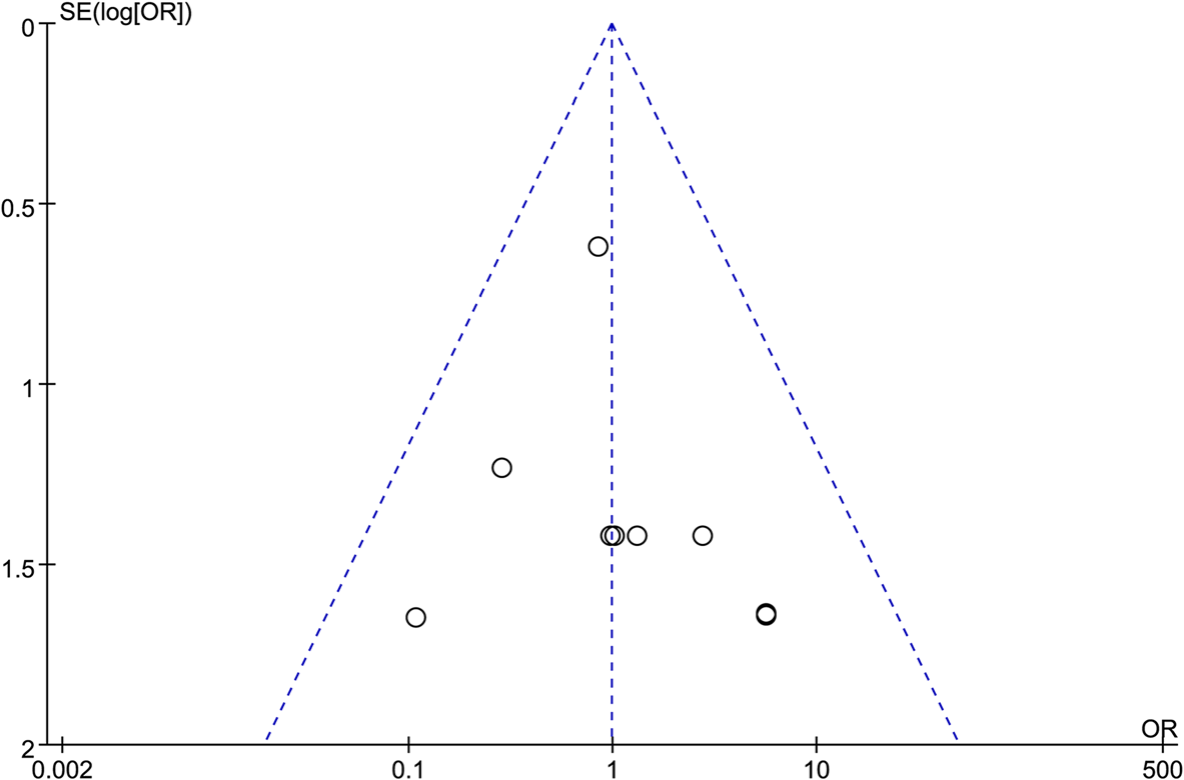
Funnel plot of ILN recurrence. OR: odds ratio

Sensitivity analysis that respectively excluded the two studies published in Chinese with English abstracts [30, 31], three studies of relatively low-quality [10, 25, 31], two studies that did not match for T category [10, 29] and three studies that did not detect LN metastasis mainly by MRI [28, 30, 31] yielded no significant changes in OS, RRFS, DFS, DMFS and ILN recurrence compared with the original meta-analysis (Table 3). All between-study heterogeneities for ILN recurrence and 3-year OS, RRFS, DFS and DMFS were non-significant (all *P* > 0.05; Table 3). Thus, the results of the comparison of ILNSI versus ILNPI were highly stable. A funnel plot of the studies included in this meta-analysis was created for ILN recurrence. All studies lay inside the 95% CIs, indicating no obvious publication bias (Fig. 4). Publication bias was not significant for OS, RRFS, DFS, DMFS and ILN recurrence (*P* = 0.985, 0.769, 0.574, 0.89, 0.848, respectively).

## Discussion

Some previous studies have reported prophylactic node irradiation of the upper neck is sufficient for patients with N0 NPC. However, these studies [11, 32] did not directly compare the efficacy of ILNSI with ILNPI; therefore, whether the efficacy of ILNSI is not inferior to ILNPI is unknown. Thus, a robust, comprehensive meta-analysis is needed to answer this issue. To the best of our knowledge, this is the first meta-analysis to compare the efficacy of ILNSI with ILNPI for patients with unilateral or bilateral neck node-negative NPC. By including nine eligible studies with a total of 2, 521 patients, this meta-analysis found no significant differences in 3-year OS, RRFS, DFS, DMFS or ILN recurrence between patients undergoing ILNSI and those undergoing ILNPI. Moreover, subgroup analysis and sensitivity analysis of ILNSI versus ILNPI showed no significant changes for any outcomes, without obvious between-study heterogeneity, indicating the results of the meta-analysis had high reliability and validity.

There are three possible reasons why ILNSI was equivalent to ILNPI. First, scientific and technological progress has reduced the value of ILNPI for unilateral or bilateral neck node-negative NPC to some extent. Widespread use of advanced imaging methods such as MRI and PET/CT has made it easier to detect LN metastases and subclinical metastases; imaging has a sensitivity of 77–88.1% for detection of LN metastases and subclinical metastases compared with 60% for palpation [33]. Thus, the risk of patients undergoing ILNSI having undetected LN metastases is low in the modern era. Also, in the IMRT era, the regional control rate is pretty high, regardless of whether ILNSI or ILNPI was employed. The 5-year RRFS rate has been reported to reach 95–98% for patients with N0–1 NPC receiving IMRT [34]. Ou et al. even reported a 5-year RRFS rate of 100% for 66 patients with only RLN metastases treated by IMRT [29]. Second, LN metastases spread in an orderly fashion from the higher to lower level LNs, for example, from the upper to lower neck, and unilateral LN metastases usually spread down from higher level LNs to the ipsilateral LNs. Also, the incidence of skip nodal metastasis is very low (0.5%) [10]. LN recurrence in the ipsilateral lower neck is rare in patients with unilateral or bilateral neck node-negative NPC. Thus, ILNSI may provide equivalent regional control as ILNPI; ILNPI could be considered unnecessary. Third, when considering quality of life, vital organs such as the thyroid gland, trachea, vessels to the irradiated area and lung apexes receive less exposure to radiation in patients undergoing ILNSI, resulting in a lower incidence of side-effects such as hypothyroidism, neck fibrosis, carotid occlusive disease, unilateral recurrent laryngeal nerve palsy and fibrosis of the lung apex [25, 27, 29]. Radiation-induced hypothyroidism had been reported in many studies, and the incidence varies from 22 to 29%, related to the irradiation volume [7, 8]. These side-effects have different degrees of impact on function, quality of life and even life expectancy [35]; this may explain why patients undergoing ILNSI in the study by Li et al. [27] had a higher 3-year OS rate than patients undergoing ILNPI (89.5% vs. 87.4%), though the difference was non-significant. Thus, ILNSI is feasible for patients with unilateral or bilateral neck node-negative NPC, as it can avoid over-treatment without compromising the regional control rate or survival outcomes.

With respect to heterogeneity, two points are noteworthy. First, in the analysis of RRFS, DFS and DMFS, the study by Chen et al. [26] favored ILNSI (HR < 1) while the remaining studies [10, 24, 25, 27–31] favored ILNPI (HR > 1). Though this trend was not significant, it indicates potential between-study heterogeneities. One probable explanation is that the ILNPI group in the study by Chen et al. [26] contained more patients with T3–4 category NPC than the ILNSI group (70 versus 32 patients). Since advanced T category is regarded as a negative prognostic factor for survival, patients receiving ILNPI may have a poorer prognosis due to their more advanced tumor stage. For OS, the study by Li et al. [27] favored ILNSI (HR < 1) while the remaining studies [10, 24–26, 28–31] favored ILNPI (HR > 1), which may result from the fact that in that study, patients treated using ILNSI had a significantly lower risk of side-effects, including lower neck grade I acute dermatitis (*P* < 0.001), lower neck grade I skin atrophy (*P* < 0.001) and fibrosis of the lung apex (*P* = 0.019) compared to patients receiving ILNPI, which may confer an improvement in OS for patients receiving ILNSI. Second, this meta-analysis included patients treated using different radiotherapy techniques, including 2D-RT, IMRT and 3D-CRT, which may lead to heterogeneity and introduce potential bias when assessing the results of treatment. Moreover, IMRT has been reported to provide a better dose distribution and improve regional control compared with 2D-RT [28]. Thus, the inequalities in radiotherapy techniques among the included studies may also influence the analysis. To consider this issue, we performed subgroup analysis according to radiotherapy techniques (i.e., IMRT and 2D-RT), which indicated ILNPI was equivalent to ILNSI with non-significant between-study heterogeneity, regardless of radiotherapy technique. However, our assessment of whether 3D-CRT leads to equivalent outcomes is crude, which may lead to between-study heterogeneity.

Several limitations of this meta-analysis must be taken into consideration. First, all studies included were retrospective, except for a single RCT with a relatively small sample size. The random sequence generation and blinding were insufficient, which reduced the quality of the studies. Second, clinical staging was based on a variety of staging systems in different studies, including the 6th and 7th editions of the American Joint Committee on Cancer. This may result in unbalanced baseline characteristics between the two comparison groups and affect the final results. Lastly, side effects and complications were not closely examined in the included studies, so we could not perform meta-analysis of side-effects and complications. Given the lack of a difference in efficacy between ILNPI and ILNSI, side-effects and complications deserve more attention; future analyses should be performed to examine this issue when sufficient additional studies are available.

## Conclusion

Our meta-analysis showed that ILNSI results in a similar ILN recurrence rate and 3-year OS, RRFS, DFS and DMFS compared to ILNPI in unilateral or bilateral neck node-negative NPC. Therefore, ILNSI is feasible for unilateral or bilateral neck node-negative NPC.

## Additional file

Additional file 1: **Table S1.** Quality assessment of eight retrospective studies using the modified Newcastle-Ottawa scale. Y = yes; N = no; U = unclear. **Table S2.** Quality assessment of one randomized controlled trial. Y = yes; U = unclear. **Figure S1.** Risk of bias graph: evaluation of risk of bias across all included studies. **Figure S2.** Risk of bias summary: evaluation of risk of bias of each included study. The green/yellow/red circles represent low/unclear/high risk of bias, respectively. (DOC 58 kb)

## Abbreviations

2D-RT: Two-dimensional radiotherapy
CI: Confidence interval
CT: Computed tomography
DFS: Disease-free survival
DMFS: Distant metastasis-free survival
ENI: Elective neck irradiation
HR: Hazard ratio
*I*^*2*^: I-square
ILN: Ipsilateral lower neck
ILNPI: Ipsilateral lower neck prophylactic irradiation
ILNSI: Ipsilateral lower neck sparing irradiation
IMRT: Intensity-modulated radiotherapy
LNs: Lymph nodes
MOOSE: Meta-analysis of Observational Studies in Epidemiology recommendations for study reporting
MRI: Magnetic resonance imaging
NPC: Nasopharyngeal cancer
OR: Odds ratio
OS: Overall survival
PRISMA: Preferred Reporting Items for Systematic Reviews and Meta-analysis
RCT: Randomized controlled trial
RLNs: Retropharyngeal lymph nodes
RRFS: Regional recurrence-free survival
SCF: Supraclavicular fossa
WHO: World Health Organization
WNI: Whole neck irradiation
*χ*^*2*^: Chi-square

## References

[1] Wei WI, Sham JS (2005). Nasopharyngeal carcinoma. Lancet (London, England).

[2] Lee AW, Foo W, Law SC, Poon YF, Sze WM, Sk O (1997). Nasopharyngeal carcinoma: presenting symptoms and duration before diagnosis. Hong Kong Med J.

[3] Ng WT, Lee AWM, Kan WK, Chan J, Pang ESY, Yau TK (2007). N-staging by magnetic resonance imaging for patients with nasopharyngeal carcinoma: pattern of nodal involvement by radiological levels. Radiother Oncol.

[4] Lee AWM, Lin JC, Ng WT (2012). Current management of nasopharyngeal cancer. Seminars in radiation oncology.

[5] Lee AW, Sham JS, Poon YF, Ho JH (1989). Treatment of stage I nasopharyngeal carcinoma: analysis of the patterns of relapse and the results of withholding elective neck irradiation. Int J Radiat Oncol Biol Phys.

[6] Lee N, Harris J, Garden AS, Straube W, Glisson B, Xia P (2009). Intensity-modulated radiation therapy with or without chemotherapy for nasopharyngeal carcinoma: radiation therapy oncology group phase II trial 0225. J Clin Oncol Off J Am Soc Clin Oncol.

[7] Zhai RP, Kong FF, Du CR, Hu CS, Ying HM (2017). Radiation-induced hypothyroidism after IMRT for nasopharyngeal carcinoma: clinical and dosimetric predictors in a prospective cohort study. Oral Oncol.

[8] Lin Z, Wang X, Xie W, Yang Z, Che K, Wu VW (2013). Evaluation of clinical hypothyroidism risk due to irradiation of thyroid and pituitary glands in radiotherapy of nasopharyngeal cancer patients. J Med Imaging Radiat Oncol.

[9] Sham JS, Choy D, Wei WI (1990). Nasopharyngeal carcinoma: orderly neck node spread. Int J Radiat Oncol Biol Phys.

[10] Tang L, Mao Y, Liu L, Liang S, Chen Y, Sun Y (2009). The volume to be irradiated during selective neck irradiation in nasopharyngeal carcinoma: analysis of the spread patterns in lymph nodes by magnetic resonance imaging. Cancer.

[11] Gao Y, Zhu G, Lu J, Ying H, Kong L, Wu Y (2010). Is elective irradiation to the lower neck necessary for N0 nasopharyngeal carcinoma?. Int J Radiat Oncol Biol Phys.

[12] Lee AWM, Ng WT, Hung WM, Choi CW, Tung R, Ling YH (2009). Major late toxicities after conformal radiotherapy for nasopharyngeal carcinoma-patient- and treatment-related risk factors. Int J Radiat Oncol Biol Phys.

[13] Fu J, Zhou JY, Chong VF, Khoo JB (2013). Indication of lower neck irradiation in nasopharyngeal carcinoma without nodal metastasis: the potential impact of tumor volume. Chin Med J.

[14] Liberati A, Altman DG, Tetzlaff J, Mulrow C, Gotzsche PC, Ioannidis JP (2009). The PRISMA statement for reporting systematic reviews and meta-analyses of studies that evaluate healthcare interventions: explanation and elaboration. BMJ.

[15] Stroup DF, Berlin JA, Morton SC, Olkin I, Williamson GD, Rennie D (2000). Meta-analysis of observational studies in epidemiology: a proposal for reporting. Meta-analysis Of Observational Studies in Epidemiology (MOOSE) group. JAMA.

[16] Tierney JF, Stewart LA, Ghersi D, Burdett S, Sydes MR (2007). Practical methods for incorporating summary time-to-event data into meta-analysis. Trials.

[17] Green S. Cochrane handbook for systematic reviews of interventions: Cochrane book series[J]. Naunyn-Schmiedebergs Archiv für experimentelle Pathologie und Pharmakologie. 2008;5(2):S38.

[18] Wells GA, Shea BJ, O'Connell D, Peterson J, Welch V, Losos M (2014). The Newcastle–Ottawa Scale (NOS) for assessing the quality of non-randomized studies in meta-analysis. Appl Eng Agric.

[19] Taggart DP, D'Amico R, Altman DG (2001). Effect of arterial revascularisation on survival: a systematic review of studies comparing bilateral and single internal mammary arteries. Lancet (London, England).

[20] Parmar MK, Torri V, Stewart L (1998). Extracting summary statistics to perform meta-analyses of the published literature for survival endpoints. Stat Med.

[21] Parmar MKB, Torri V, Stewart L, Parmar MKB, Torri V, Stewart L (1999). Extracting summary statistics to perform meta-analyses of the published literature for survival endpoints. Stat Med.

[22] Higgins JP, Thompson SG, Deeks JJ, Altman DG (2003). Measuring inconsistency in meta-analyses. Br Med J.

[23] DerSimonian R, Laird N (1986). Meta-analysis in clinical trials. Control Clin Trials.

[24] Tang LL, Tang XR, Li WF, Chen L, Tian L, Lin AH (2017). The feasibility of contralateral lower neck sparing intensity modulation radiated therapy for nasopharyngeal carcinoma patients with unilateral cervical lymph node involvement. Oral Oncol.

[25] Zeng L, Sun XM, Chen CY, Han F, Huang Y, Xiao WW (2014). Comparative study on prophylactic irradiation to the whole neck and to the upper neck for patients with neck lymph node-negative nasopharyngeal carcinoma. Head Neck.

[26] Chen M, Tang LL, Sun Y, Mao YP, Li WF, Guo R (2014). Treatment outcomes and feasibility of partial neck irradiation for patients with nasopharyngeal carcinoma with only retropharyngeal lymph node metastasis after intensity-modulated radiotherapy. Head Neck.

[27] Li JG, Yuan X, Zhang LL, Tang YQ, Liu L, Chen XD (2013). A randomized clinical trial comparing prophylactic upper versus whole-neck irradiation in the treatment of patients with node-negative nasopharyngeal carcinoma. Cancer.

[28] Sun JD, Chen CZ, Chen JZ, Li DS, Chen ZJ, Zhou MZ (2012). Long term outcomes and prognostic factors of n0 stage nasopharyngeal carcinoma: a single institutional experience with 610 patients. Asian Pac J Cancer Prev.

[29] Ou X, Shen C, Kong L, Wang X, Ding J, Gao Y (2012). Treatment outcome of nasopharyngeal carcinoma with retropharyngeal lymph nodes metastasis only and the feasibility of elective neck irradiation. Oral Oncol.

[30] Xie FY, Peng M, Hu WH, Han F, Wang X, Xu HM (2010). Prophylactic irradiation of cervical lymph nodes for stage-N0 nasopharyngeal carcinoma. Chin J Cancer.

[31] Li Y, Cao KJ, Chen QY, Xie GF, Huang PY (2005). Radiotherapy on neck for nasopharyngeal carcinoma patients with negative cervical lymph node. Chin J Cancer.

[32] Hu W, Zhu G, Guan X, Wang X, Hu C (2013). The feasibility of omitting irradiation to the contralateral lower neck in stage N1 nasopharyngeal carcinoma patients. Radiat Oncol.

[33] Mw VDB, Castelijns JA, Croll GA, Stel HV, Valk J, van der Waal I (1991). Magnetic resonance imaging vs palpation of cervical lymph node metastasis. Arch Otolaryngol Head Neck Surg.

[34] Su SF, Han F, Zhao C, Chen CY, Xiao WW, Li JX (2012). Long-term outcomes of early-stage nasopharyngeal carcinoma patients treated with intensity-modulated radiotherapy alone. Int J Radiat Oncol Biol Phys.

[35] Ho FC, Tham IW, Earnest A, Lee KM, Lu JJ (2012). Patterns of regional lymph node metastasis of nasopharyngeal carcinoma: a meta-analysis of clinical evidence. BMC Cancer.

