# Supplementary material for: Feasibility of ipsilateral lower neck sparing irradiation for unilateral or bilateral neck node-negative nasopharyngeal carcinoma: systemic review and meta-analysis of 2, 521 patients
Source: Radiat Oncol. 2018 Aug 6;13:141. doi: 10.1186/s13014-018-1087-x (PMC6080384; doi:10.1186/s13014-018-1087-x)
Supplement: Supplementary file 1 — Table S1. Quality assessment of eight retrospective studies using the modified Newcastle-Ottawa scale. Y = yes; N = no; U = unclear. Table S2. Quality assessment of one randomized controlled trial. Y = yes; U = unclear. Figure S1. Risk of bias graph: evaluation of risk of bias across all included studies. Figure S2. Risk of bias summary: evaluation of risk of bias of each included study. The green/yellow/red circles represent low/unclear/high risk of bias, respectively. (DOC 58 kb) [file 13014_2018_1087_MOESM1_ESM.doc]

| **Table S1.** Quality assessment of eight retrospective studies using the modified Newcastle-Ottawa scale   | First author /year | Selection | | |  | Comparability | |  | Outcome | | Quality score | | --- | --- | --- | --- | --- | --- | --- | --- | --- | --- | --- | | Assignment for treatment | Representative treatment group | Representative reference group |  | Comparable for primary items | Comparable for secondary items |  | Assessment for outcome | Adequate follow-up |  | | Chen/2014 [26] | Y/Y | Y/Y | Y/Y |  | N/N | Y/Y |  | Y/Y | Y/Y | ★★★★★★★★ | | Xie/2010 [30] | Y/Y | Y/Y | Y/Y |  | N/N | Y/Y |  | Y/Y | Y/Y | ★★★★★★★★ | | Ou/2012 [29] | Y/Y | Y/Y | Y/Y |  | Y/Y | Y/Y |  | Y/Y | Y/Y | ★★★★★★★★★ | | Tang/2017 [24] | Y/Y | Y/Y | Y/Y |  | Y/Y | Y/Y |  | Y/Y | N/N | ★★★★★★★★ | | Zeng/2014 [25] | Y/Y | Y/Y | Y/Y |  | N/N | Y/Y |  | Y/Y | N/N | ★★★★★★★ | | Li/2005 [31] | Y/Y | Y/Y | Y/Y |  | N/N | Y/Y |  | Y/Y | N/N | ★★★★★★ | | Tang/2009 [10] | Y/Y | Y/Y | Y/Y |  | N/N | U/Y |  | Y/Y | N/N | ★★★★★★ | | Sun/2012 [28] | Y/Y | Y/Y | Y/Y |  | N/N | Y/Y |  | Y/Y | Y/Y | ★★★★★★★★ |   Abbreviations: Y = yes; N = no; U = unclear. |
| --- | --- | --- | --- | --- | --- | --- | --- | --- | --- | --- | --- | --- | --- | --- | --- | --- | --- | --- | --- | --- | --- | --- | --- | --- | --- | --- | --- | --- | --- | --- | --- | --- | --- | --- | --- | --- | --- | --- | --- | --- | --- | --- | --- | --- | --- | --- | --- | --- | --- | --- | --- | --- | --- | --- | --- | --- | --- | --- | --- | --- | --- | --- | --- | --- | --- | --- | --- | --- | --- | --- | --- | --- | --- | --- | --- | --- | --- | --- | --- | --- | --- | --- | --- | --- | --- | --- | --- | --- | --- | --- | --- | --- | --- | --- | --- | --- | --- | --- | --- | --- | --- | --- | --- | --- | --- | --- | --- | --- | --- |

**Table S2. Quality assessment of one randomized controlled trial**

| First author/  year | Adequate random sequence generation | Allocation concealment | Double blinding  method | Adequate assessment  of each outcome | Avoid selective outcome  reporting | Handing of  missing data |
| --- | --- | --- | --- | --- | --- | --- |
| Li/2013 [27] | Y/Y | Y/Y | U/Y | Y/Y | Y/Y | Y/Y |

Abbreviations: Y = yes; U = unclear.


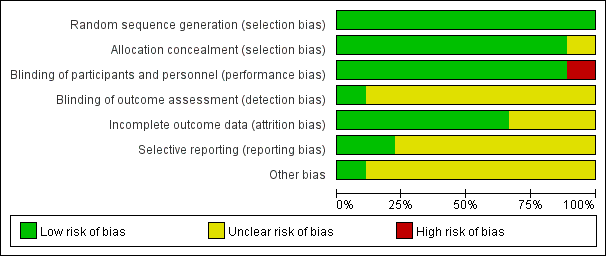


**Figure S1.** Risk of bias graph: evaluation of risk of bias across all included studies.

**
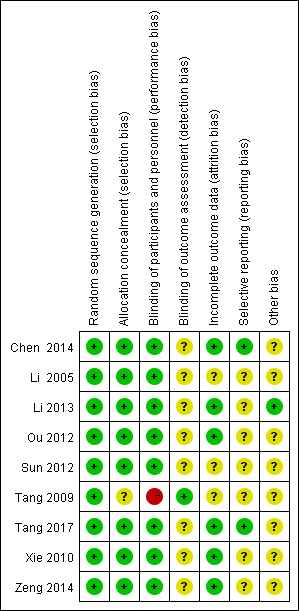
**

**Figure S2.** Risk of bias summary: evaluation of risk of bias of each included study. The green/yellow/red circles represent low/unclear/high risk of bias, respectively.
